# Re-examining extreme sleep duration in bats: implications for sleep phylogeny, ecology, and function

**DOI:** 10.1093/sleep/zsac064

**Published:** 2022-03-13

**Authors:** Christian D Harding, Yossi Yovel, Stuart N Peirson, Talya D Hackett, Vladyslav V Vyazovskiy

**Affiliations:** Department of Physiology Anatomy and Genetics, Sir Jules Thorn Sleep and Circadian Neuroscience Institute, University of Oxford, Oxford, UK; The Kavli Institute for Nanoscience Discovery, Oxford, UK; School of Zoology, Faculty of Life Sciences, Tel-Aviv University, Tel-Aviv, Israel; Sagol School of Neuroscience, Tel-Aviv University, Tel-Aviv, Israel; The Kavli Institute for Nanoscience Discovery, Oxford, UK; Nuffield Department of Clinical Neurosciences, Sir Jules Thorn Sleep and Circadian Neuroscience Institute, University of Oxford, Oxford, UK; Department of Zoology, University of Oxford, Oxford, UK; Department of Physiology Anatomy and Genetics, Sir Jules Thorn Sleep and Circadian Neuroscience Institute, University of Oxford, Oxford, UK; The Kavli Institute for Nanoscience Discovery, Oxford, UK

**Keywords:** Chiroptera, sleep, torpor, social behavior

## Abstract

Bats, quoted as sleeping for up to 20 h a day, are an often used example of extreme sleep duration amongst mammals. Given that duration has historically been one of the primary metrics featured in comparative studies of sleep, it is important that species specific sleep durations are well founded. Here, we re-examined the evidence for the characterization of bats as extreme sleepers and discuss whether it provides a useful representation of the sleep behavior of Chiroptera. Although there are a wealth of activity data to suggest that the diurnal cycle of bats is dominated by rest, estimates of sleep time generated from electrophysiological analyses suggest considerable interspecific variation, ranging from 83% to a more moderate 61% of the 24 h day spent asleep. Temperature-dependent changes in the duration and electroencephalographic profile of sleep suggest that bats represent a unique model for investigating the relationship between sleep and torpor. Further sources of intra-specific variation in sleep duration, including the impact of artificial laboratory environments and sleep intensity, remain unexplored. Future studies conducted in naturalistic environments, using larger sample sizes and relying on a pre-determined set of defining criteria will undoubtedly provide novel insights into sleep in bats and other species.

**Statement of Significance**

Bats are suggested to be amongst the longest sleeping animals to regularly feature in comparative analyses of sleep. However, considerable variation in daily sleep duration has been reported between species of Chiroptera and within species measured at different ambient temperatures. We discuss the significance of this variation for the characterization of bats as extreme sleepers and reaffirm the importance of measuring sleep duration in a way that is accurate, reproducible and capable of capturing natural differences in sleep between species. Furthermore, by highlighting unique aspects of their sleep, including the potential co-occurrence of torpor, we hope to raise the status of bats as a group in which to study sleep phylogeny, ecology and function.

## Introduction

Sleep is increasingly being recognized as a state with cognitive and restorative functions that are of vital importance in the lives of all animals [1]. In bats (Order: Chiroptera), a diverse group of mammals loosely separated into large frugivorous and small insectivorous forms, sleep has received little attention. Till date the sleep patterns of only four species of bats have been characterized using electrophysiological recordings (see Table 1). Notwithstanding, bats are well represented in the sleep literature because of the daily duration of their sleep. Research articles, textbooks, and popular science books have propagated the view that bats are extreme sleepers, spending up to 20 h a day in this state [2–11]. This is based on the sleep behavior research of two species, the little brown bat (*Myotis lucifugus*) and the big brown bat (*Eptesicus fuscus*) [12, 13]. Despite the importance of these studies to the overall characterization of bats as extreme sleepers, few have attempted to contextualize their findings. In particular, the sparse accounts of the conditions used in electrophysiological experiments and the lack of diversity of species studied has not been addressed.

**Table 1.** Sleep architecture and constitutive variables for bat species in which electrophysiology has been performed

Suborder	Family	Species	Common name	Habitat	Diet	Lifespan (years)	Adult mass (g)	Total sleep time (h)	NREM time (h)	REM time (h)	NREM- REM cycle time (min)
Yang.^i^	Vesp.^ii^	*Myotis lucifugus*	Little brown bat	Temperate	Insectivorous	34	8	19.92	17.93(.90)^iii^	1.99(.10)	12.0
Yang.	Vesp.	*Eptesicus fuscus*	Big brown bat	Temperate/ Tropical	Insectivorous	19	16	19.70	15.80(.80)	3.90(.20)	7.5
Yin.^iv^	Ptero.^v^	*Cynopterus sphinx*	Greater short- nosed fruit bat	Tropical	Frugivorous	10	44	14.83	13.68(.92)	1.15(.08)	6.1
Yin.	Ptero.	*Eonycteris spelaea*	Cave nectar bat	Tropical	Frugivorous/ Nectarivorous	-	57	14.73	13.94(.95)	0.86(.06)	2.2
^i^Yangochiroptera		^ii^Vespertilionidae		^iii^Proportion of total sleep		^iv^Yinpterochiroptera			^v^Pteropodidae		

Sleep data from [6, 12, 13]. Mass data from [14]. Lifespan data from AnAge [15].

Why some animals sleep longer than others remains a leading question in the sleep research field. Comparative studies use the variation in sleep duration across animal species to search for correlates that may give insight into the functions of sleep [16–18]. Because the number of species available for such studies is relatively small, extreme durations such as those documented in bats can have a strong influence on the results and subsequent conclusions. It is therefore important to understand the factors that explain the extreme sleep durations recorded in bats and whether they have been correctly compared with other species.

Here, we first present the case for bats displaying an extreme daily sleep duration in comparison to other species. We then discuss challenges to this assessment posed by conflicting data and confounding factors in the original data. From the case of bats, we extrapolate general issues associated with the sleep duration dataset which may have contributed to the mixed success of the comparative method in identifying sleep functions. We end by discussing the potential contribution bats could make to the sleep field moving forward and suggest best practices for achieving this. This review reveals the complexity involved in answering as seemingly simple a question as ”how long do bats sleep?” and identifies behavioral and physiological factors that make this group an interesting case study for researching sleep function.

## Evidence of Extreme Sleep Duration

Bats have long been recognized for their propensity for sleep. An early encyclopedia entry from 1797 documents how bats “inhabit dark places, which they quit only for nocturnal excursions” and may be found “for the greatest part of the day” suspended by their feet, concealed within their wings [19]. One of the first attempts to quantify the length of sleep in bats can be found in the writings of Moffat [20] who described the “remarkably somnolent” lesser noctule (*Nyctalus leisleri*), quoting a daily sleep duration of 21.5 h during its “season of activity”, thus distinguishing the behavior from hibernation.

Emergence profiles of bat colonies provide insight into the roosting behavior and therefore, indirectly, sleep behavior of bats. The great majority of bats are nocturnal, emerging from their day roosts at dusk and returning before dawn [21]. For most species, this means movement outside the roost is restricted to between 1800–0600 or less [21, 22]. Following an initial peak in emergence activity at the beginning of the night, a secondary peak prior to dawn is also present for many species and is associated in particular with insectivory [21]. Roosting behavior may be affected by seasonal changes in temperature and day length. The time pallid bats (*Antrozous pallidus*) spend in the day roost ranges from 14 h in the summer to 19 h in the spring [23]. Furthermore, individuals may spend longer periods in the day roost than the colony emergence profile would suggest. Trident bat (*Asellia tridens*) colonies continue to make foraging trips for up to 10 h after sunset, yet individuals on average only spend around 4 h foraging per night [24]. To rest and decrease energy costs, bats spend as much as 75% of the time away from the day roost in night roosts [25, 26]. Night-roosting may therefore make a meaningful contribution to the total sleep duration in some species. Kunz [27] estimated that *M. lucifugus* spend 15 h in day roosts and 5 h in night roosts per 24 h period, with the remaining 4 h taken up by foraging.

Although studies of wild activity patterns do not differentiate between roosting behaviors (e.g. sleeping, grooming, mating), and therefore cannot be used to assess how much of this time is dedicated to sleep, they do show that bats are often in an appropriate environment to sleep for 12–20 h a day.

High temporal resolution laboratory recordings of activity facilitate finer-grain descriptions of bat rest-activity cycles. As predicted from wild observations, bats maintained under controlled 12:12 light-dark cycles largely limit their activity to the dark period [21]. This behavior has been shown to be endogenously controlled in *M. lucifugus* and tricolored bats (*Pipistrellus subflavus*), persisting over multiple days in constant darkness before beginning to free-run [28]. In the same study, bats maintained under dim light conditions were inactive for up to 18.8–20.2 h per day and exhibited the aforementioned bimodal activity pattern typical of some insectivorous species in the wild with dusk and dawn peaks and little activity in between (Supplementary Material 1). These results suggest individual bats may only be truly active for a portion of the night, providing additional time for sleep to occur.

While the length of time animals spend inactive can be used to estimate sleep time, this methodology often leads to overestimation as sleep cannot be distinguished from other quiescent states such as quiet wakefulness and torpor, though as we will see this issue also applies to more sophisticated sleep measures [29]. Behavioral definitions of sleep typically also include the assumption of a species-specific posture, reversible perceptual disengagement from the environment and a homeostatic sleep rebound following deprivation [30–32]. An alternative approach used in most modern sleep studies is to rely on well-founded electrophysiological correlates of behavioral sleep [30]. Electrical activity in the brain is detected by electrodes and converted into an electroencephalogram (EEG) from which signals characteristic of behaviorally defined sleep and wake can be identified. When performed in conjunction with electromyography (EMG), this approach allows researchers to divide the time course of sleep into two main stages, rapid eye movement (REM) and nonrapid eye movement (NREM) sleep, based on muscle tone and the frequency composition of the EEG signal.

An electrophysiological approach was first applied to the study of sleep behavior in bats by D. R. Brebbia and associates in the 1960s and 70s [12, 33].^.^ Regional brain EEG and nuchal EMG were performed in tandem with measurements of brain temperature and heart rate in chronically implanted animals. In the first of two conference abstracts, the posture and diurnal distribution of sleep is described for two species of vespertilionids native to the Americas, *E. fuscus* and *M. lucifugus*, followed by a detailed electrophysiological characterization of different brain regions during sleep [33] (Box 1). As in other animals, sleep presented behaviorally as physical quiescence in a stereotyped posture and could be separated into two stages with distinct electrophysiological profiles. Namely, NREM cortical EEG was characterized by high voltage slow rhythms and spindle activity, and REM cortical EEG by a desynchronized trace similar to wakefulness. The second conference abstract describes the effects of ambient temperature manipulations on sleep in *M. lucifugus* [12]. At 33 °C, 83% of the recording time was occupied by REM or NREM sleep, which equates to a total daily sleep duration of 19.9 h, the figure most often quoted for this species. Interestingly, neither of these studies mention the duration of sleep for *E. fuscus*. The earliest record for this species is in a review by Zepelin and Rechtschaffen [13], quoting a sleep duration of 19.7 h. The citation for this data is a personal correspondence from Brebbia dated to the same period as the recordings in *M. lucifugus* which could suggest that sleep duration was also measured in *E. fuscus*. However, as there is no mention of this in Brebbia’s writings [12, 33], the provenance of this measurement cannot be verified.

The daily sleep durations measured using electrophysiological criteria appear to confirm that bats are indeed sleeping during the long periods of time they spend inactive. Whether such sleep durations should be considered extreme depends on comparison with other animals. In one review of sleep across mammalian orders, measures of daily sleep duration in Chiroptera were estimated to be two standard deviations above the mean (µ = 10.46, σ = 4.95) and more than 4 h greater than the next longest order [34]. On this basis, a strong argument can be made for bats being considered extreme sleepers.

## A Re-examination of Bat Sleep Duration

### Variation in Chiroptera

In contrast to the narrative so far presented, not all measurements of sleep duration in bats may be classed as extreme by mammalian standards. 40 years after the first electrophysiological measurements of bat sleep duration, Zhao et al. [6] recorded sleep electrophysiology in the cave nectar bat (*Eonycteris spelaea*) and the greater short-nosed fruit bat (*Cynopterus sphinx*). Five to six mixed sex adult specimens of each species were captured from wild populations and transferred to a temperature and light regime controlled laboratory setting. As in the work of Brebbia et al. [12, 33], EEG and EMG electrodes were implanted to measure brain and muscle activity respectively and the sleep–wake cycle was scored using standard mammalian criteria. In stark contrast to the *M. lucifugus*, Zhao et al. [6] reported sleep durations well below 20 h in both species (see Table 1 for a comparison of sleep architecture). On an average *C. sphinx* slept 14.8 h per day, which was accumulated predominantly during the light period of the 12:12 h light–dark regime employed in the experiment. The average daily sleep duration was slightly lower for *E. spelaea* at 14.7 h, and unlike *C. sphinx* was split evenly between the light and dark periods.

Zhoa et al.’s [6] findings represent a marked departure from previous sleep duration measures. Based on their findings alone, bats would appear to have daily sleep durations comparable to species like the Syrian hamster (14.4 h) [35] that do not generally enter discussions of extreme sleep. Furthermore, there is no obvious difference in the methodology of their experiment that may have contributed to sleep duration; they used wild-caught animals kept in laboratory conditions and scored sleep using standard electrophysiological criteria. Nonetheless, it may be possible to reconcile these differences in sleep duration between Zhoa et al.’s [6] study and the work of Brebbia and Pyne [12] if we consider the choice of species.

The species used in the two electrophysiological studies of chiropteran sleep duration have distinct evolutionary histories. *M. lucifugus* and *Eptesicus fuscus* are vespertilionids belonging to the suborder Yangochiroptera, whereas *E. spelaea* and *C. sphinx* are pteropodids belonging to the suborder Yinpterochiroptera. Molecular phylogenetics suggests the two chiropteran suborders diverged in the early Paleogene and have been evolving in tandem for 64 million years [36]. In addition to their phylogenetic separation, the species used in the studies also differ in their basic biology. The vespertilionids are a widespread family of small insectivores which typify the common chiropteran assemblage [37]. On the other hand, the predominantly frugivorous pteropodids (fruit bats) are larger and more reliant on vision for orientation than other bats as most lack the ability to echolocate [38, 39].

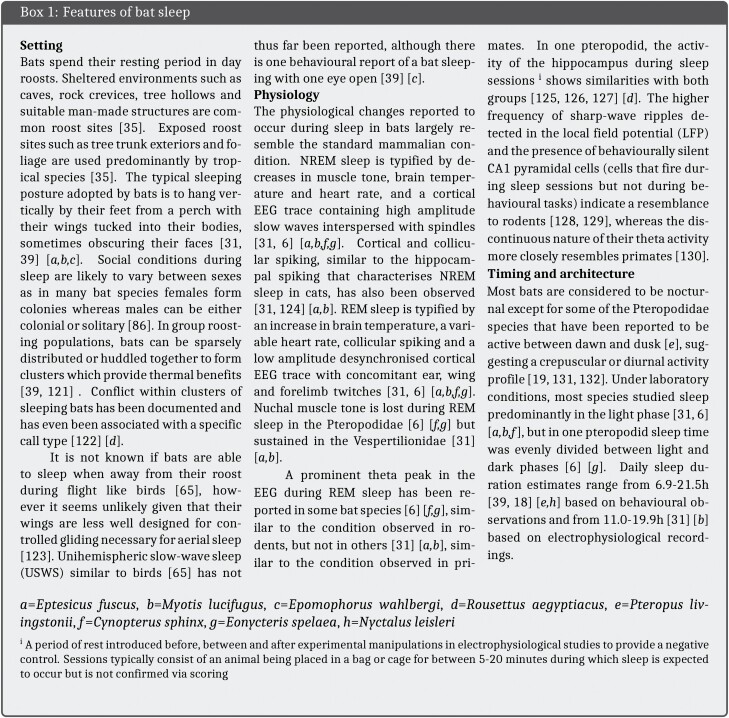

Given the variation between the species used in these studies, it is perhaps not surprising that sleep duration measures also differed. In fact, there is an evidence to support the hypothesis that sleep duration differs between pteropodids and other bat species. In their investigation into daily temperature changes of bats, Burbank and Young [40] noted the peculiarity that attendants at two captive fruit bats colonies in the United Kingdom had never observed them “fully asleep”. This may be an exaggeration of the observation that fruit bats are more active during the day than their counterparts. For example, using behavioural criteria Downs et al. [41] found that nocturnal Wahlberg’s epauletted fruit bats (*Epomophorus wahlbergi*) slept for only 28.8% of the 12 h light phase in the laboratory and were usually awake when observed in the wild during the same period. Using similar criteria, the large flying fox (*Pteropus vampyrus*) was estimated to sleep up to 71% of the 12 h light phase on average between the sexes; however, this still represents a decrease compared with *M. lucifugus* and does not take into account the lower sleep propensity expected of nocturnal animals in the dark phase [42].

If sleep duration is lower in the Pteropodidae, this would be consistent with comparative studies of sleep in mammals that show large, herbivorous species tend to sleep less than small, carnivorous species [3, 13, 16, 17, 34]. One theory to explain this relationship, the energy conservation hypothesis, posits that if the function of sleep is energy conservation, small mammals with a high metabolic rate may have evolved to sleep longer than large mammals with lower metabolic rates [43]. In support of this hypothesis, pteropodids are almost exclusively homeothermic with heterothermy reported in only a few small species [44], suggesting a lower requirement for energy conservation [37].

An alternative to the energy conservation hypothesis is the foraging time hypothesis which posits that because sleep requires immobility, sleep duration should be negatively correlated with foraging time [34]. As a result, larger animals with higher energetic requirements and animals that consume low energy foods should sleep less. This could explain why extreme sleep durations have not been recorded in the larger, frugivorous pteropodids. However, both frugivores and insectivores spend a majority of their active phase roosting [26, 45] and have comparable foraging behavior in terms of duration and distance travelled [46] which suggests foraging time may not be an explanatory factor for differences between pteropodids and other bats.

One final explanation for pteropodids sleeping for shorter durations than other bats could be related to sociality. Mammals that sleep socially have been found to have reduced sleep durations compared with species that sleep alone, perhaps as a result of needing to fulfil social interaction quotas [17]. A similar relationship may exist within Chiroptera, in which the highly social pteropodids [42, 47, 48] devote more time to interacting with conspecifics and less time to sleep than species such as *M. lucifugus*. The foremost problem with this theory is that most laboratory sleep studies have been conducted with isolated subjects and therefore differences between species related to their social sleeping conditions may not have been expressed. To reveal such differences will require the introduction of sleep experiments conducted under naturalistic social conditions.

Evidence from pteropodids points to there being considerable interspecific variation in sleep duration between species of bat. Whilst this in itself does not challenge the idea that some bats are capable of extreme sleep duration, it does make clear the fact that, as for any group of animals, the characterization of chiropteran sleep behavior should be informed by a range of species that encompass the order’s diversity in morphology, ecology and evolutionary history.

### Caveats to recordings of extreme sleep duration in bats

Whilst multiple sources indicate that bats may be inactive for up to 20 h a day [27, 28], only Brebbia and Pyne’s [12] work with *M. lucifugus* suggests this time is spent sleeping. Thus, the extreme sleep duration characterization depends on a single study using an unknown number of specimens. Furthermore, descriptions of most of the experimental conditions under which *M. lucifugus* were studied are limited or missing from the cited source material [12]. Because of the age of the study, the use of invasive EEG implants and reference to temperature manipulations, we can assume that Brebbia and Pyne’s sleep experiments were conducted under laboratory conditions. In general, laboratories are simple environments that fail to recreate the natural challenges to which animals in the wild have adapted and can introduce artificial challenges of their own [49]. Factors such as photoperiod [50, 51], light intensity [50], ambient temperature [52], diet [53], social environment [54] and predation risk [55–57] have all been identified as capable of influencing sleep but can be difficult to reproduce in captivity. Two of these factors in particular, ambient temperature and social environment, may have influenced the sleep behavior of bats in Brebbia and Pyne’s [12] experiments.

#### Temperature dependency and torpor

**Figure 1.**
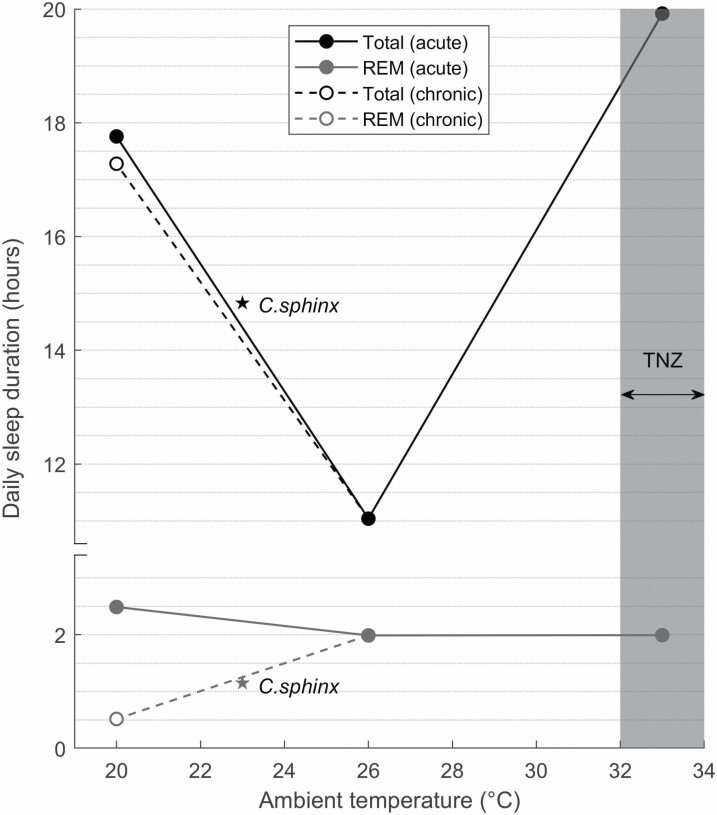
Daily sleep duration as a function of ambient temperature in the little brown bat (*Myotis lucifugus*). The 19–21°C temperature condition is represented by the mean (20°C). Hours of total sleep and REM sleep are shown both for acute exposures to all temperatures and a multi-day chronic exposure at 19–21°C. Overlap with the species specific thermoneutral zone (TNZ) at which captive bats were reported by Stones [83] to defend a homeothermic body temperature is highlighted in grey. Total sleep and REM sleep durations of *Cynopterus sphinx* are overlayed. Data for *Myotis lucifugus* adapted from Brebbia and Pyne [12] and for *Cynopterus sphinx* from Zhao et al. [6].

A notable caveat to the 19.9 h daily sleep duration reported for *M. lucifugus*, which citing studies usually ignore, is that it is temperature dependent. Brebbia and Pyne [12] characterized the sleep–wake cycle of this species when exposed to a range of ambient temperatures (Figure 1). Total sleep duration was found to vary from a maximum of 19.9 h at 33°C to a minimum of 11.0 h at 26°C. Below 19°C, EEG signals lacked discernible sleep rhythms and at 5°C became isotonic. In addition to temperature, exposure time also had an effect on sleep duration, most notably the duration of REM sleep which decreased from 2.5 h to 0.5 h following “chronic” exposure to a temperature range of 19–21°C for multiple days. This means that depending on ambient temperature, total sleep duration and REM duration can be longer or shorter than *E. spelaea* (Figure 1). Whilst it is not unusual for sleep duration to fluctuate with ambient temperature [58, 59], the magnitude of change recorded in *M. lucifugus* stands out. For example, time spent in NREM sleep increased by only 25% in mice between 26°C and 34°C [60] compared with 98% in Brebbia and Pyne’s study [12]. The extreme temperature dependence displayed by *M. lucifugus* and the finding that they do not exhibit an extreme sleep duration across all temperatures raises two questions. Firstly, for which temperature condition should sleep duration be reported? Secondly, are durations derived from temperature dependent sleep recordings comparable with other animals?

To answer the first question, we must consider the thermal conditions faced by bats in their natural habitats. Bats are found in all environments apart from certain deserts and high latitudes [37] and have been recorded at environmental temperatures as low as −17° ^◦^C during the winter and as high as 55°C during the summer ([61, 62] reviewed in [63]). The temperature at which bats sleep is influenced by their roosting behavior. Generally, roosts provide stable microclimates relative to outside air temperatures and have been shown to increase in temperature by as much as 7°C when occupied by clusters of bats because of social thermoregulation [64]. As a result, even in temperate climates during the summer with an average ambient air temperature of 18°C, average day roosts temperatures can exceed 35°C [65]. These observations suggest that the 5–33°C temperature range used by Brebbia and Pyne [12] is biologically relevant. It seems appropriate to report sleep duration from an ambient temperature at which sleep rhythms were clearly identifiable, which leaves both the 26°C and 33°C conditions. By this logic both 19.9 h and 11.0 h are equally characteristic of daily sleep duration in this species. Such a dramatic change in sleep duration in response to natural environmental variation, in this case in ambient temperature, is not specific to *M. lucifugus* (see [66, 67]) and species-specific sleep durations are likely to be context dependent in general. Nevertheless, using a single figure to quantify *M. lucifugus* sleep duration in Brebbia and Pyne’s [12] study is not representative of their findings unless qualified with reference to ambient temperature.

Answering the second question is more difficult as there are many reasons why sleep duration may be dependent on temperature. One possibility is that sleep duration in these experiments was influenced by torpor. Like many small endotherms [68–71], bats can become heterothermic to alleviate the high energetic demands of maintaining a constant body temperature. This results in the expression of torpor, an energy conserving physiological state in which metabolic rate is decreased and body temperature, ventilation and heart rate are depressed [72, 73]. Conditions believed to promote entry into torpor include low environmental temperatures, limited food availability and drought [74]. The temperature threshold at which torpor becomes favorable seems to be particularly high among bats compared with other mammals, with some reported to become heterothermic at temperatures of *>*30°C) [24], and is likely as a result of their high rates of thermal conductance [75] combined with a more metabolically costly form of locomotion [76]. Thus, although torpor is often associated with winter hibernation (seasonal torpor) in response to low environmental temperatures, many bats in all climes also employ shorter bouts of torpor (daily torpor) throughout the year when energy supply is low [77]. In the case of *M. lucifugus*, individuals frequently become heterothermic, with torpor bouts lasting 2–3 weeks during the winter and 1–24 h during the summer [78].

As in other animals, the relationship between sleep and torpor in bats is insufficiently understood [79]. Functionally, these states are thought to be distinguishable. For instance, the energy allocation model posits that sleep optimizes the allocation of energy for biological activities not required during wake through state-dependent metabolic partitioning whereas torpor serves to minimize total energy investment through metabolic rate reduction [80]. Hence bats would be expected to switch between strategies depending on whether biological investment (sleep) or energy conservation (torpor) is the prevailing requirement. Nevertheless, it is possible for these states to coincide ([69, 81, 82] see Box 2). If sleep and torpor did coincide in Brebbia and Pyne’s [12] recordings, this may render their findings incompatible with sleep recordings from other animals for two reasons. First, sleep in a state of torpor may not be comparable with nontorpid sleep. An increase in slow-wave activity (i.e., incidence, amplitude) has been observed during the period following a torpor bout similar to that seen after sleep deprivation [83, 84]. This suggests that the rate at which the function of sleep is fulfilled, referred to as sleep intensity and modelled by slow-wave activity in mammals, during torpid sleep is insufficient to meet the body’s demands [83–85]. Thus similar lengths of torpid and nontorpid sleep are not comparable in function. Second, sleep duration may be decoupled from sleep need in species capable of torpor. As torpor is typically entered through NREM sleep [86], animals may sleep to fulfil their torpor requirement. In studies where torpor cannot be distinguished from sleep, the occurrence of torpor will therefore result in the elongation of recorded sleep duration.

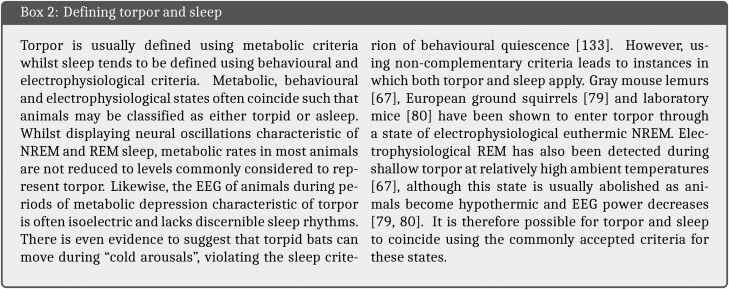

As torpor is primarily a measure of dealing with a low energy supply rather than cold defense [73], the cost of defending a homeothemic body temperature at both high and low ambient temperatures could favor the expression of daily torpor bouts and by association sleep depending on energy status. Without body temperature or metabolic measures, we can only infer whether torpor occurred in Brebbia and Pyne’s [12] study. Sleep at 19–21°C was characterized by an increase in the ratio of NREM:REM sleep and a reduction in the voltage of electrical activity in the brain which could indicate shallow torpor. The induction of shivering at this temperature range could indicate bursts of maintenance and rewarming thermogenesis during torpor-arousal cycles [82]; however, important details of the time course of shivering are absent. Below 19°C an isoelectric EEG lacking sleep rhythms suggests a deepening of torpor, which the authors recognized as a “unique state of consciousness...at hypothermic extreme” [12]. Although the sleep EEG was described as “typically mammalian” above 26°C and into the thermoneutral zone (TMZ) of *M. lucifugus* (32–37°C) [87], the possibility that bats entered torpor cannot be ruled out at these temperatures either given that normal sleep rhythms are present during high temperature torpor bouts in other animals and that bats have been reported to be torpid above 30°C in the wild [24, 69]. Therefore, it is possible that the longer sleep durations measured by Brebbia and Pyne [12] may represent a combination of both sleep and daily torpor.

In summary, the 19.9 h sleep duration recorded at a temperature of 33°C is not representative of the sleep behavior of the *M. lucifugus* in Brebbia and Pyne’s [12] study. Sleep duration varied considerably across a naturalistic range of temperatures for this species. Furthermore, the influence of torpor on sleep duration could not be accounted for. In fact this holds for any study of sleep in bats that lacks information regarding the temperature or metabolic state of the subjects, including the study by Zhao et al. [6]. Therefore, whilst *M. lucifugus* are capable of extreme sleep durations, such lengths are not the common condition and their sleep may not be directly comparable with other animals in which sleep has been studied.

#### Social environment

Of the laboratory conditions that are thought to have an effect on sleep, one stands out as having the potential to influence bats more than many other mammalian orders: the social environment. Most bats are gregarious, which has been attributed in part to a limited availability of suitable roost sites, and are found in groups ranging in size from several individuals to some of the largest aggregations of mammals in the world [88, 89]. Social roosting can provide benefits such as reduced thermoregulatory costs (social thermoregulation) and reduced evaporative water loss simply as a result of aggregation [64]. Furthermore, many groups of bats contain complex social systems in which animals engage in a diversity of social behaviors [88, 90–92]. *M. lucifugus* form stable colonies (i.e. that persist throughout the reproductive season) of many thousands of individuals usually in permanent roosts such as caves and mines [93–95]. Although it has been suggested that social systems of *M. lucifugus* colonies are relatively simple in comparison to some bat species, evidence of social behaviors such as vocal communication during interactions, swarming and even social learning indicate that the social environment plays an important role in the lives of these animals [96–98]. Unfortunately, we do not know the specific housing conditions used by Brebbia and Pyne [12]. However, most animals, whether social or solitary in the wild, are isolated during sleep studies [49]. Thus, it is important to consider the possibility that the sleep data for *M. lucifugus* were not collected under naturalistic social conditions.

Multiple relationships between sleep duration and sociality have been suggested. A key benefit of grouping which is thought to influence sleep is decreased risk of predation. For example, theoretical and empirical evidence suggests that the collective vigilance of groups increases with group size even if individual vigilance is reduced [99, 100]. As predation risk is negatively correlated with sleep duration across mammals, this could suggest that grouping could increase sleep duration [17]. Commensurate with this, Lendrem [101] found that as corporate vigilance in Barbary doves (*Streptopelia risoria*) increased with group size, so too did the time individuals spent with their eyes closed which could suggest an increase in sleep duration. In fact, the opposite trend has been observed in comparative studies of sleep which find greater sleep durations in solitary versus social species [17]. To explain this trend, it has been suggested that sleep may be disrupted in social environments, either because of individuals spending more time undertaking social interactions [17] or being disturbed by conspecifics.^6^ It should be noted that as most “normative” sleep duration data used in comparative studies has been acquired from isolated animals regardless of their grouping behavior in the wild, differences in sleep between species associated with sociality may not have been expressed, making it difficult to predict the effects of the social environment. For example, REM sleep episode duration is increased in colony-forming rock hyraxes (*Procavia capensis*) when housed with conspecifics compared with isolation [102].

One final possibility which has received little attention is that sleep duration is increased by grouping but this effect is not observed in comparative studies because of the aforementioned isolation of subjects in sleep experiments. Without a group to sleep in, social animals may be extra vigilant in sleep experiments, causing a negative bias in the sleep duration data for these species.

Although the effect of the social environment on sleep has not been studied directly in bats, there is indirect evidence to support the theory that sociality may influence sleep through predation risk. Klose et al. [103] recorded vigilance behavior in a colony of adult tree-roosting flying foxes (*Pteropus poliocephalus*) and found that bats at the periphery of the colony engaged in more environmental vigilance events (vigilance directed towards the surroundings rather than conspecifics) and were more vigilant in general than centrally positioned bats, though only the first result was significant. Although predation is likely to be more relevant to open roosting bats, even cave dwelling species such as *M. lucifugus* experience predation in their roosting environment, meaning the social environment may still influence predation risk [93, 104]. Indeed, activity levels have been found to differ significantly with position in clusters of *M. lucifugus*, with central individuals spending close to 4% more of the day roosting period at rest than peripheral individuals [65]. Whether these group position effects are related to corporate vigilance or other factors such as social thermoregulation has yet to be determined.

On the other hand, there is also indirect evidence in bats to suggest sleep may be disrupted by conspecifics. Individual actions such as grooming have been reported to disturb neighbors and aggressive interactions with physical and auditory components have been documented between roosting bats [93, 105]. Such interactions may be concentrated at the onset of the roosting period when arriving bats compete for positions within clusters [65]. Groups of *M. lucifugus* have been observed exiting torpor canonically in “arousal cascades” [106, 107]. Because the social thermoregulation benefits of arousing in a cascade are less than arousing synchronously, it has been suggested that such events result from maladaptive disturbances of torpid individuals by normothermic individuals [107]. This is supported by evidence that a torpor arousal cascade initiated by researchers at a cave-dwelling colony of northern myotis bats (*Myotis septentrionalis*) and *M. lucifugus* bats continued long after the researchers had left [108]. If bats do respond to social arousal cues, this could explain how bats trapped inside caves lacking diurnal environmental rhythms are able to maintain their circadian phase relative to free-moving bats in the same roost [109, 110].

Both extension and reduction of sleep duration may be predicted outcomes of sleeping socially in bats. The opposing forces of reduced predation risk and increased disruption risk associated with grouping may even act in tandem, with the overall effect on sleep depending on the balance between the two. For example, grouping may promote sleep in species that form small clusters and use exposed roost sites, whilst the opposite may occur in species that form large aggregations in protected roost sites. To add an additional layer of complexity, the influence of such effects in laboratory sleep experiments is likely dependent on the specific social conditions used, as suggested by the case of the rock hyrax [102]. It is therefore difficult to predict the effect of the social environment in recordings of extreme sleep duration in *M. lucifugus*. However, it seems likely that the natural sleep behavior of bats in the wild is influenced by the social environment meaning characterizations of their sleep behavior should reflect this.

## Implications for Comparative Analyses of Sleep Duration

Arguably the ultimate reason we are interested in measuring parameters of sleep in different species is to gain insight into the functions of this phenomenon, and one of the most important parameters historically has been sleep duration [30, 111]. It is usually assumed that sleep confers some form of benefit, the magnitude of which is a function of the time spent in this state. However, there are also significant costs associated with sleep, such as the inability to access sustenance and reproduce [2]. As sleep and wake are generally mutually exclusive states, with some notable exceptions, such as unihemispheric sleep in birds [67, 112] and marine mammals [113, 114], we would expect natural selection to act on the trade-off between the benefits and costs of each state to optimize sleep duration [80]. The existence of interspecific variation in sleep duration is important as it suggests that differences between species alter the optimal trade-off between time spent sleeping and awake. Thus, if we can identify the features that are important in predicting differences in sleep duration between species, we might be able to infer something about the functions of sleep [111]. This has been the aim of cross-species comparative studies which correlate sleep parameters and other potentially relevant features of morphology (e.g., body mass), physiology (e.g., metabolic rate), and ecology (e.g., predation risk). A number of theories of sleep function have subsequently been developed from this methodology. For example, the energy conservation hypothesis for sleep was first posited by Allison and Van Twyver [8] based on comparisons of NREM sleep duration in endotherms and ectotherms.

Overall, however, efforts to correlate interspecific variation in sleep duration with features thought to be associated with sleep function, such as basal metabolic rate (BMR), encephalization and body mass, have met with mixed success, often producing weak or inconsistent results [17, 115]. For example, it was traditionally thought that BMR was positively correlated with sleep time [13], thus giving support for an energy conservation function, until the introduction of statistical controls for shared evolutionary history suggested the inverse [111]. From our re-examination of extreme sleep duration in bats, we can infer issues with the sleep duration dataset that may explain it’s sensitivity to different applications of the comparative method.

The first inferred issue is sample size. Comparative studies in mammals typically sample only around 50–80 species or c.1% of the total number of species [13, 115]. The majority of mammalian orders (67%) [116] contain fewer than 100 species so are theoretically only represented by a single species at this level of sampling (given the idealistic assumption of random sampling). A single species can give a distorted picture of an order’s sleep behavior, as evidenced by the c.5 h range in daily sleep duration in bats. The case of Chiroptera therefore suggests that greater sampling is required to capture variation at the level of the order. Furthermore, undersampling is directly responsible for bats being considered extreme sleepers, for *M. lucifugus* and *Eptesicus fuscus* were the only chiropterans available to the initial comparisons of sleep duration amongst animals which birthed this idea. The case of Chiroptera suggests that some of the observed variation in sleep architecture between taxa could be the result of chance sampling of extreme representatives, leading to a false phylogenetic signal. If true, this would have significant implications for use of the comparative method and could explain some of its shortcomings.

The second inferred issue is the age of studies measuring sleep duration. Many studies used in comparative analyses date back more than 40 years when electrophysiological sleep research was in its nascency. Although many of the fundamental aspects of such studies have not changed since, some that can influence the measurement of sleep duration have. The paramount example of this from bats is the treatment of heterothermy, especially torpor. In extreme sleep recordings from bats, no mention is made of the possible occurrence of torpor despite changes in physiology indicative of torpor being reported. This is problematic, for sleep duration may be affected by torpor and torpid sleep may not be comparable with nontorpid sleep in terms of function. This in turn speaks to a major oversight of comparative studies: the omission of sleep intensity [111]. Just as torpid and nontorpid sleep may differ in function, so might sleep of different intensities, and should not therefore be treated equally. As our understanding of sleep function evolves, the lack of detail in the reporting of experimental variables in early experiments such as Brebbia and Pyne [12] may cause us to reassess whether they should be included in comparative analyses.

The third inferred issue is the use of laboratory conditions. Despite an increase in recent years of electrophysiological sleep studies conducted using wild mammals [56, 117–119] and birds [66, 67, 120] the vast majority of sleep data are laboratory recordings, meaning the results of comparative analyses are predicated on the assumption that animals sleep the same in the laboratory as in the wild [18]. There is some evidence to suggest that the sleep behavior of wild bats is recapitulated faithfully in a laboratory setting, such as the finding that insectivorous species exhibit bimodal activity pattern in both environments [21, 28]. However, there is also evidence to suggest that bats alter their sleep behavior in response to laboratory conditions, such as the marked differences in sleep duration of *M. lucifugus* at different ambient temperatures [12]. Furthermore, the influence of many standard laboratory conditions in sleep experiments, such as social isolation, have not been explored at all despite being known modulators of sleep duration. Variation between studies in how much laboratory conditions differ from those in which species evolved, and in the sensitivity of species to these differences, may therefore constitute important sources of variation in the sleep duration dataset with unknown effects on comparative analyses.

## Best Practices for Future Experiments

The purpose of this review is not to discourage the comparative approach or as others have to advise against the use of bats in such analyses [13]. Rather, the aim is to highlight the need for studies that measure sleep duration in a way that allows for meaningful comparison. In fact, Chiroptera may be an ideal group in which to employ the comparative approach. Bats are one of the most speciose orders of mammals, second only to rodents [121]. Furthermore, they are a diverse group, with many features important to sleep differing both within and between the two major evolutionary lineages; the Yangochiroptera and the Yinpterochiroptera. Unlike previous cross-taxa comparative analyses that suffer from the effects of confounding variables, Chiroptera potentially presents us with the opportunity to identify factors associated with specific examples of the evolution of sleep duration between species within the same order. For example, by investigating sleep in frugivorous relatives of *M. lucifugus*, it could be determined whether the shorter sleep duration of pteropodids is related to diet or evolutionary history. However, to achieve this aim, sleep duration must be measured in a way that is accurate, reproducible and capable of capturing natural differences in sleep between species.

We suggest the following as good practices for designing experiments to characterize natural sleep behavior in bats. These should not be interpreted as a checklist of necessary criteria but as a framework to help guide researchers and highlight potential factors they may not have considered which should be addressed when interpreting the results of an experiment.

In an ideal scenario, such experiments should be carried out in the natural environment in which that bat’s sleep behavior has evolved [49]. Given the high risk, high reward nature of these studies, researchers may wish to sacrifice some elements of optimal experimental design (e.g., control of environmental conditions, large sample sizes) to acquire these data. In such cases, detailed accounts of the methods and conditions under which experiments were performed are paramount. Where experiments must be carried out in a laboratory, all attempts should be made to replicate the conditions in which that bat would sleep in the wild. This includes both abiotic (e.g., temperature, humidity, light levels, and regimes) and biotic (e.g., group size, food type, and availability) conditions. Studies should be appropriately powered by recording multiple individuals over multiple sleep–wake cycles. Recordings should only commence after subjects have been habituated to the experimental conditions and if using invasive monitoring techniques, after the effects of surgical procedures have dissipated. To assess sleep duration, both behavioral and electrophysiological correlates of vigilance states should be measured and validated through assessment of homeostatic sleep regulation and state-specific arousal thresholds, particularly when studying a species for the first time. Sleep rhythms should be detected using cranial EEG electrode positions (e.g. frontal and occipital cortex, cerebellar reference) informed by the underlying brain structure and should be measured in tandem with EMG and EOG to facilitate sleep scoring. Some measurement of the metabolic state of the bats during sleep experiments such as body temperature, heartrate or O^2^ consumption should be recorded to assess potential crossover with periods of torpidity. Behavioral features that correlate strongly with electrophysiological sleep state or duration in a species should be noted as these may be useful for measuring sleep in subsequent studies where more invasive techniques are difficult to employ (e.g. wild studies).

We also suggest the following considerations be made when analyzing and reporting data from sleep experiments in bats. When scoring vigilance states in a species of bat for the first time, researchers should not assume that states will present in the same way as other mammals and carry the burden of proof to show that their scoring is justified through statistical means or by presenting appropriate examples of the features associated with each state. Researchers should comment on any occurrences of marked metabolic depression that could constitute torpor and whether they coincided with sleep. Homeostasis should be confirmed using NREM slow wave activity to model sleep intensity. Alternative metrics of sleep intensity should be sought in behavioral studies, such as sleep continuity [122]. A clear record of all conditions used in sleep experiments should be reported to ensure that readers can contextualize the results and if needed reproduce them. Finally, researchers should report both the variability in daily sleep duration of bats in the study as well as the average used to characterize their sleep behavior.

## Conclusion

The extreme sleep durations reported in some bat species have potentially important implications for our understanding of sleep function. However, there is a weak body of available evidence to support the characterization of bats as extreme sleepers. We have identified multiple methodological and theoretical caveats to existing sleep measurements, such as the unknown influence of torpor and social environment. Furthermore, reports of shorter sleep durations in other species of bat could represent a challenge to this characterization. These points may reflect general issues with the quantity and quality of sleep duration data available to comparative analyses of sleep. If we are to answer the question of whether bats are extreme sleepers, further recordings of sleep duration are needed. If extreme sleep durations cannot be replicated, this would suggest that sleep duration in bats has been exaggerated. If extreme durations can be replicated, this would confirm that interspecific differences in sleep duration exist between bats and would identify Chiroptera as a group in which to explore questions about the factors responsible for the variation in sleep duration amongst animals.

## Supplementary Material

zsac064_suppl_Supplementary_Material
