# Supplementary material for: Re-examining extreme sleep duration in bats: implications for sleep phylogeny, ecology, and function
Source: Sleep. 2022 Mar 13;45(8):zsac064. doi: 10.1093/sleep/zsac064 (PMC9366634; doi:10.1093/sleep/zsac064)
Supplement: zsac064_suppl_Supplementary_Material [file zsac064_suppl_supplementary_material.docx]

Re-examining extreme sleep duration in bats: implications for sleep phylogeny, ecology and

Function: Supplementary Materials

1. - Christian D. Harding, Sir Jules Thorn Sleep and Circadian Neuroscience Institute, Department of Physiology Anatomy and Genetics, University of Oxford, Oxford, UK

The Kavli Institute for Nanoscience Discovery, Oxford, UK

1. - Yossi Yovel, School of Zoology, Faculty of Life Sciences, Tel-Aviv University, Tel-Aviv, Israel

Sagol School of Neuroscience, Tel-Aviv University, Tel-Aviv, Israel

1. - Stuart N. Peirson, Sir Jules Thorn Sleep and Circadian Neuroscience Institute, Nuffield

Department of Clinical Neuroscience, University of Oxford, UK

The Kavli Institute for Nanoscience Discovery, Oxford, UK

1. - Talya D. Hackett, Department of Zoology, University of Oxford, Oxford, UK
2. - Vladyslav V. Vyazovskiy, Sir Jules Thorn Sleep and Circadian Neuroscience Institute,

Department of Physiology Anatomy and Genetics, University of Oxford, Oxford, UK

The Kavli Institute for Nanoscience Discovery, Oxford, UK

## Supplementary 1: Kymography analysis

Inactivity times were estimated from the published graphical representations of the kymograph recordings made by Griffin and Welsh [S1] (Supplementary figure 1). Image processing software was used to calculate the cumulative proportion of inactive periods in each 24h kymography plot. Only continuous activity periods were included (i.e., single movements were ignored). Bats in this experiment were hand-fed once daily, during which time the kymography record is void. We calculated a sleep time range to account for the possibility that bats would have been sleeping (maximum) or awake (minimum) during void periods had they not been interrupted for feeding. *Myotis lucifugus* sleep duration was measured from the kymography plot of a single bat over 3 days (15h00-14h59, 12-15th June 1936) under subdued daylight conditions (Sunrise=19h20,Sunset=04h10). *Pipistrellus subflavus* sleep duration was measured from the kymography plot of a single bat over 10 days (12h00-11h59, 16-21;22-25;26-28th October 1936) in constant darkness (excluding feeding periods). We ignored days where Griffin and Welsh^S1^ attributed high activity to dehydration. Kymography plots for a second pipistrelle are also available, however the recording periods are not contiguous and therefore cannot be directly compared with the other recordings.

## Supplementary 2: Literature search

Due to the limited number of sources, we did not perform a systematic review of the literature. Collations of mammalian sleep parameters were identified by searching JSTOR with the key words “mammalian” and “sleep” and those containing reference to bats were identified. Original sources of information pertaining to bats were then found by tracing citations. Information regarding sleep in bats not contained in summary articles were found by searching JSTOR with the key words “bat” and “sleep”.

# Supplementary figure caption

**Supplementary figure 1:** Kymography analysis example. In Griffin and Welsh’s^S1^ figures, each vertical black bar represents an activity bout where height denotes activity level and width denotes duration. Single vertical lines represent single movements. The horizontal black line is broken where recordings were interrupted (e.g. by feeding). Each 24 hour trace was sectioned into activity bouts (animals active), sleep bouts (animals inactive or single movements) and void bouts (no recording). Calculations for minimum and maximum sleep time estimated from these figures are shown.

**Supplementary figure 1**


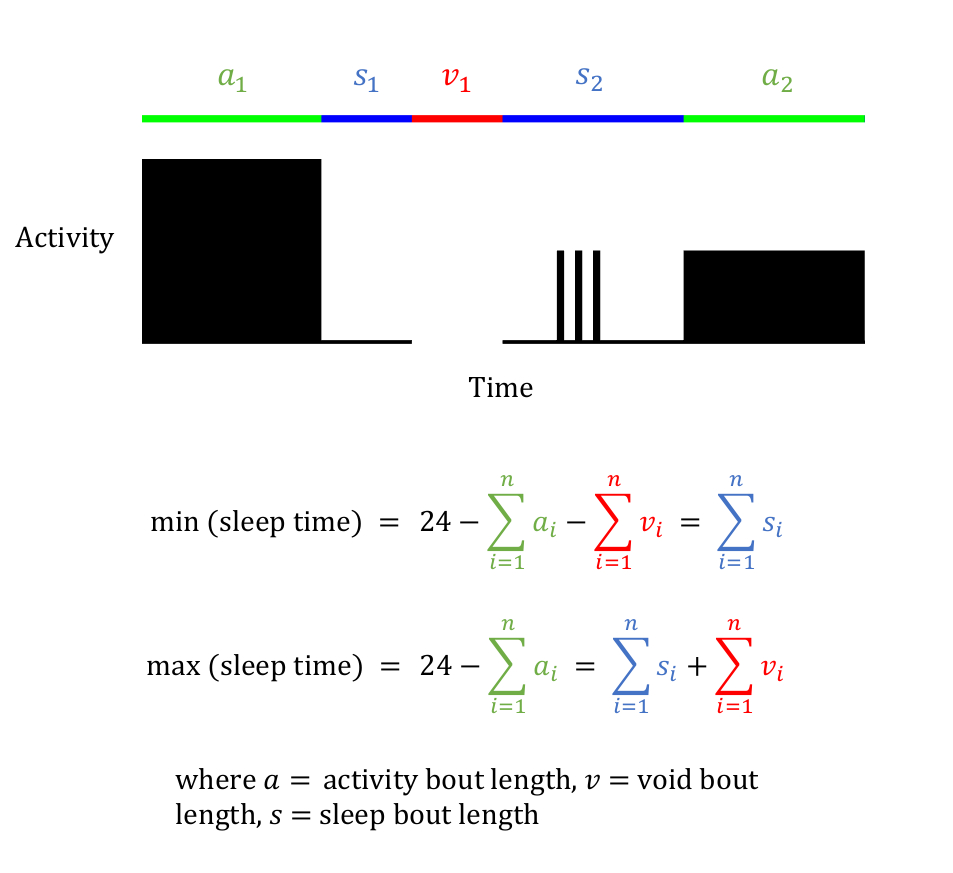


**Supplementary references:**

^[S1]^ Griffin DR, Welsh JH. Activity Rhythms in Bats under Constant External Conditions. J Mammal. 1937;18(3):337.
